# The Vascular Microenvironment in Glioblastoma: A Comprehensive Review

**DOI:** 10.3390/biomedicines10061285

**Published:** 2022-05-31

**Authors:** Alejandra Mosteiro, Leire Pedrosa, Abel Ferrés, Diouldé Diao, Àngels Sierra, José Juan González

**Affiliations:** 1 Department of Neurosurgery, Hospital Clínic de Barcelona, 08036 Barcelona, Spain; abferres@clinic.cat (A.F.); jjgonzal@clinic.cat (J.J.G.); 2 Laboratory of Experimental Oncological Neurosurgery, Hospital Clínic de Barcelona, 08036 Barcelona, Spain; lepedrosa@clinic.cat (L.P.); diao@clinic.cat (D.D.); asierrajim@gmail.com (À.S.); 3 Department of Medicine and Life Sciences (MELIS), Universitat Pompeu Fabra, 08003 Barcelona, Spain

**Keywords:** glioblastoma, tumor microenvironment, perivascular niche, angiogenesis, neovascularization, organoids, 3D models, anti-angiogenic therapy

## Abstract

Glioblastoma multiforme, the deadliest primary brain tumor, is characterized by an excessive and aberrant neovascularization. The initial expectations raised by anti-angiogenic drugs were soon tempered due to their limited efficacy in improving the overall survival. Intrinsic resistance and escape mechanisms against anti-VEGF therapies evidenced that tumor angiogenesis is an intricate multifaceted phenomenon and that vessels not only support the tumor but exert indispensable interactions for resistance and spreading. This holistic review covers the essentials of the vascular microenvironment of glioblastoma, including the perivascular niche components, the vascular generation patterns and the implicated signaling pathways, the endothelial–tumor interrelation, and the interconnection between vessel aberrancies and immune disarrangement. The revised concepts provide novel insights into the preclinical models and the potential explanations for the failure of conventional anti-angiogenic therapies, leading to an era of new and combined anti-angiogenic-based approaches.

## 1. Introduction

Glioblastoma multiforme, the most common and the deadliest primary brain tumor, is characterized by its high vascularization and by the overexpression of proangiogenic factors as it is one of the most vessel-rich tumors [1,2]. The standard of treatment has remained unchanged for more than two decades [3], meanwhile survival is still very limited [4]. The initial excitement brought by the introduction of anti-angiogenic drugs within the therapeutic armamentarium was soon mitigated due to their poor efficacy in improving overall survival [5]. Those initial treatment regimens were meant to target a specific pathway in the neovascularization process, yet the tumor somehow managed to escape this aggression and even became more aggressive [6,7,8]. Since then, our knowledge about the tumor microenvironment (TME) composition and behavior has evolved substantially, making it clear that multitarget therapies are crucial in confronting such a heterogeneous and dynamic entity [9].

This holistic review covers the essentials of the vascular microenvironment of glioblastoma, starting from the perivascular niche and its elements, dealing with the vascular generation processes and related signaling pathways, understanding the endothelial–tumor interaction and how it encourages tumor spread along the vessels, and ending with the interconnection between vessel aberrancies and immune disarrangement. The revised concepts will provide novel insights into the preclinical models and the modern designs and their current limitations when emulating the vascular microenvironment. Ultimately, vascular-targeted therapeutic strategies will be discussed, acknowledging the reasons for previous failure and heading towards new and combined anti-angiogenic-based approaches.

## 2. The Perivascular Niche

**Figure 1:**
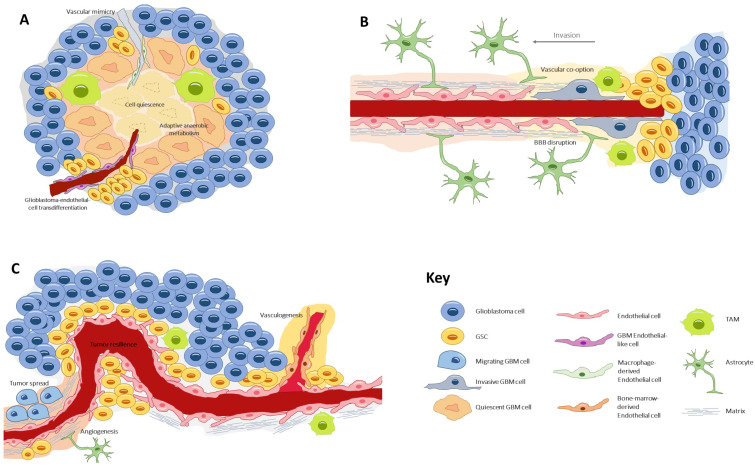
The three types of glioblastoma stem cell microenvironments: (**A**) the perivascular niche, where GSCs lie in close contact with the endothelium, a favorable ambient that promotes tumor resilience and proliferation; (**B**) the necrotic niche, at the tumor core, where hypoxia and lack of nutrients drive the formation of new vessels by diverse processes. Under these undesirable conditions, cells develop adaptive anaerobic metabolism and quiescence; (**C**) the invasive front, at the tumor margin, where GSCs exhibit an infiltrative nature and make use of preexisting healthy vessels to spread throughout the healthy parenchyma. GSC, glioblastoma stem cell; TAM, tumor-associated macrophage.

The glioblastoma microenvironment is composed of the noncancerous cells, the extracellular matrix, and the biomolecules residing inside the tumor, all of which establish complex interactions with the tumor cells and modulate genetic and epigenetic expression. In this landscape, a remarkable type of cells resides, the glioblastoma stem cells (GSCs) [10], a subpopulation which is capable of self-renewal and pluripotent differentiation and is responsible for invasion, chemo/radio resistance, and relapse [11,12,13]. Three habitats have been described for these cells, each one determining different environment-driven behaviors (Figure 1).

First is the *perivascular niche* [14], where GSCs lie in close contact with the endothelium, a favorable ambient that promotes tumor resilience and proliferation [15]. Second is the *necrotic niche* [16,17], at the tumor core, where hypoxia and lack of nutrients drive the formation of new vessels by diverse processes. Under these undesirable conditions, cells develop adaptive anaerobic metabolism and quiescence [18,19]. Third is the *invasive front* [20], at the tumor margin, where GSCs exhibit an infiltrative nature and make use of preexisting healthy vessels to spread throughout the parenchyma [21].

The perivascular niche is a privileged environment allowing tumor-initiating cell maintenance, endothelial and tumor cell crosstalk, the promotion of vascular development and perivascular tumor spread [22]. Indeed, the perivascular niche has been designated as one of the reasons for treatment resistance and tumor recurrence [23]. Progressing with this concept, Hira et al. [24,25] introduced the idea of the periarteriolar niche, seeing that GSCs were predominantly clustered around arterioles. This specification of vessel type distribution helped explain some microenvironmental properties. Arterioles, contrary to capillaries, are transport and not exchange vessels, and thus promote a peri-hypoxic area rich in hypoxia-inducible factor (HIF)-1α and vascular endothelial growth factor (VEGF). In addition, arterioles have an adventitia harboring mesenchymal stem cells and thus may harbor a natural habitat for GSCs.

Later, Kumar et al. described a position-dependent tumor cell behavior, determined by the relative distance of the tumor cell to a vessel, a gradient that they called *metabolic zonation* [26]. Proximity in the perivascular tier determined a distinctive mammalian target of rapamycin (mTOR)-derived anabolic metabolism in the tumor cell, which resulted in increased aggressiveness and resistance to chemo- and radiotherapy. Therefore, vessels shape the TME in a gradient-like configuration. In this model, the relative distance of a tumor cell from the nearest vessel is a crucial determinant of the exposure to oxygen, nutrients, and paracrine signals and governs the metabolic activity of the tumor and the resistance to conventional therapies [26]. This idea of a perfusion gradient within the tumor replaces the classic dichotomous conception of “normoxic” versus “hypoxic” microenvironments [27].

## 3. Microvascular Patterns

The niche concept in malignant gliomas was originally conceived to describe the sites where GSCs reside within the tumor bulk and where the TME exerts its maximal influence [28].

Notably, the perivascular niche is not a homogeneous construction. The glioblastoma intratumoral diversity accounts for a variety of microvascular patterns, as first described by Birner et al. [29]. Four types of microvessel conformations mold the interaction between glioma cells, endothelial cells, pericytes, astrocytes, and macrophages, an interaction that influences the genetic expression and tumor behavior and ultimately determines the clinical outcomes. Birner’s classical patterns, revisited by Chen et al. [30], include the following:

### 3.1. Microvascular Sprouting

Microvascular sprouting comprises capillary vessels with a healthy appearance, similar to those seen in physiological angiogenesis. This classical vascular pattern is associated with better survival rates.

### 3.2. Vascular Cluster

Vascular clusters are aggregations of more than two vessels, without connective stroma between them.

### 3.3. Vascular Garland

The vascular garland is a circular or spiral arrangement of intertwined aberrant vessels, frequently distributed around the necrotic core.

### 3.4. Glomeruloid Vascular Proliferation

The glomeruloid vascular arrangement comprises a cluster of more than two vessels encased by connective stroma, resembling a glomerular structure.

The vascular garland and the glomeruloid proliferation are bizarre vascular patterns, associated with high proliferative tumors, in which cell growth seems to outpace neovascularization. Notably, in both of these vessel patterns, the cell bundles co-express alpha-Smooth Muscle Actin (α-SMA) or Nestin, which suggests that the pericytes belong to the transdifferentiation of GSCs [31]. All in all, it could be argued that glioblastomas with orderly vascular patterns are less aggressive and rely mostly on classic angiogenic processes; thereupon, they are likely to profit more from anti-angiogenic therapy than their cluttered counterparts, in which fast growth and necrosis promotes unconventional VEGF-independent neovascularization [29].

In addition to the vascular organization pattern, another appealing subdivision of tumor vasculature distinguishes between tumor-associated and tumor-derived vessel populations [32]. In a murine immune-competent model, the tumor-derived vessels were composed of two distinct endothelial subpopulations, exhibiting either a pro-vascular enriched profile or a pro-inflammatory lymphatic signature. This distinct molecular signature in tumor vessels might depict emerging immune phenotypes and explain the interactions between endothelium and tumor-associated macrophages, including immunosuppressive responses. Of note, both the tumor-associated and the tumor-derived vessel seemed different to the healthy endothelium, encouraging the idea of selectively targeting tumor vessels, while sparing normal endothelium [32].

## 4. Vascular Generation and Related Processes

Healthy brain vessels egress as a consequence of tumor invasion and neovascularization. Initially, normal vessels are well conformed in the so-called neurovascular unit. Here, endothelial cells are strongly entwined by tight junctions and attached to the extracellular matrix; upon the matrix, a sheath of abundant pericytes surrounds the basal lamina; the outer coverage is composed of astrocytic end-feet [33]. This cytoarchitecture corresponds to the blood-brain barrier (BBB), an extremely selective multilayered membrane that controls blood–brain product exchange.

However, as the tumor grows and vascularizes, the vessels become disorganized, dilated, and tortuous [34,35]; the BBB increases its permeability and promotes interstitial edema and immune cell invasion [36].

**Figure 2:**
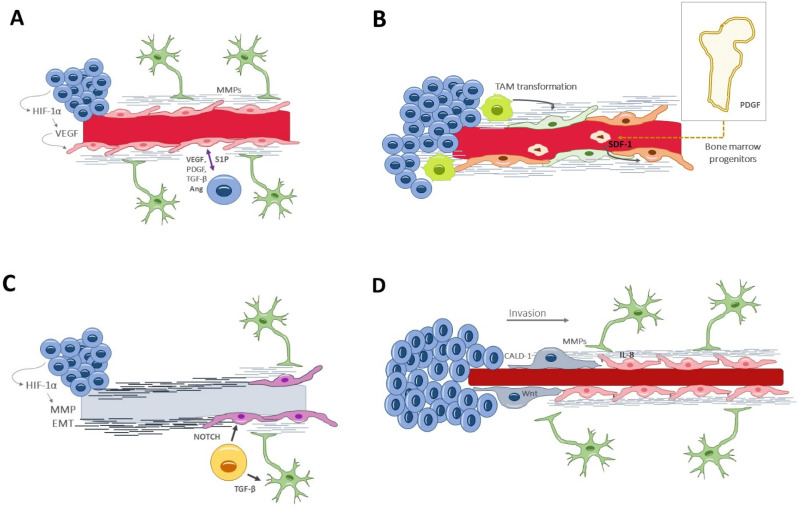
Vascular generation and related processes: (**A**) angiogenesis, the formation of new vessels from pre-existing ones. In glioblastoma, it appears in a late stage of tumor development and within the necrotic niche. (**B**) Vasculogenesis, recruiting circulating cells into neoformed vessels, relies on bone marrow-derived progenitors or tumor-associated macrophages that differentiate into endothelial cells. It happens in late stages of tumor evolution. (**C**) Vascular mimicry consists of neoformation of vessel-resembling structures that lack endothelial cells. Glioblastoma-endothelial-cell transdifferentiation is complementary to vascular mimicry and may provide a pseudoendothelial coverage to the otherwise acellular tubular structures. (**D**) Vascular co-option is a process of tumor invasion along the basolateral surface of healthy vessels. Thus, this process happens in the invasive front, in the early oncologic phase. Ang, angiopoietin; CALD1, caldesmon; EMT, epithelial–mesenchymal transition factor; HIF-1α, hypoxia-inducible factor; Il-8, interleukin-8; MMPs, matrix metalloproteinases; PDGF, platelet-derived growth factor; TGF-β, S1P, Sphingosine-1-phosphate; Transforming growth factor β; VEGF, vascular endothelial growth factor.

Traditionally, the formation of new vessels within the tumor was denoted as the sole process of angiogenesis. In recent years, several other processes have been unveiled [37], which either coexist or precede angiogenesis, and which occur at different stages of tumor development in response to specific environmental needs (Figure 2).

### 4.1. Angiogenesis

Angiogenesis [38] is the formation of new vessels from pre-existing ones; it occurs in both physiological and pathological conditions, and it is governed by an equilibrium of proangiogenic and inhibiting factors. In glioblastoma, angiogenesis seems to appear in a late stage of tumor development and within the necrotic niche. When the excessive tumor growth surpasses the vessels’ capacity to supply the metabolic demands, hypoxia acts as a potent angiogenic trigger via the HIF-1α, which induces the transcription of VEGF [39]. Angiogenesis begins with the dissolution of the extracellular matrix and the endothelial attachments, thanks to the secretion of metalloproteinases by the endothelial cells and pericytes [20]. This proteolytic degradation is followed by endothelial cell remodeling and specialization into tip and stalk types, determined by a finely synchronized interaction between Delta-like 4/NOTCH and VEGF/VEFGR2 signaling ways [40]. The process ends by recruiting pericytes that constitute the capillary sheath and by secreting basal membrane components, all of which is induced by platelet-derived growth factor receptor and angiopoietin Tie-2 receptor [41]. (Figure 2A).

### 4.2. Vasculogenesis

Vasculogenesis is yet another mechanism of new vessel formation, relying on bone marrow-derived endothelial progenitor cells [42,43]. This process of recruiting circulating cells into neoformed vessels happens in the late stages of tumor evolution (Figure 2B). Additionally, tumor-associated macrophages could also differentiate into endothelial cells for neovascularization purposes [44].

### 4.3. Vascular Mimicry

Vascular mimicry consists of the neoformation of vessel-resembling structures that lack endothelial cells. Their appearance is similar to capillaries, with a tubular wall of matrix components and a lumen where fluids and blood cells circulate [45]. This alternative mechanism of vascularization is seen in advanced and aggressive gliomas, in areas under severe hypoxia [46]. In fact, HIF-1α promotes the expression of several molecules directly implicated in the mimicry, including VEGF, matrix metalloproteinases (MMPs), TGF-β, and epithelial–mesenchymal transition factor (EMT) [47]. Liu et al. demonstrated the clinical relevance of vascular mimicry, correlating it with higher tumor grades and shorter overall survival times [48] (Figure 2C).

### 4.4. Glioblastoma-Endothelial-Cell Transdifferentiation

The pluripotency of GSCs can overcome the lack of endothelial cells or circulating progenitors by direct transdifferentiation into endothelial-like cells [49]. This mechanism is complementary to the vascular mimicry and may provide a pseudoendothelial coverage to the otherwise acellular tubular structures. Again, hypoxia is the main driver for transdifferentiation, in a way that does not involucrate VEGF but, rather, the NOTCH signaling path [50] (Figure 2C), a highly conserved transmembrane plasma receptor that mediates cell–cell communication during myogenic progression and self-renewal.

### 4.5. Vascular Co-Option (or Angiotropism or Perivascular Migration)

Vascular co-option [51,52,53] is an angiogenic-related phenomenon by which the tumor cells make use of already existing vessels and which does not imply the formation of new ones. It is actually a process of tumor invasion, alternative to the white matter spread, by which glioma cells travel along the basolateral surface of the vessels and ultimately incorporate these healthy vessels into the tumor. Thus, this process happens in the invasive front and in the early oncologic phase and is VEGF-independent [54,55]. Importantly, this infiltrative process requires astrocyte displacement, contributing to vessel disarrangement and BBB leakage [56]. Specific tumor–endothelium interactions are needed, which will be discussed later in the text (Figure 2D).

Vessel co-option has been proposed as an intrinsic or acquired resistance mechanism to anti-angiogenic therapy [57]. Actually, the oligodendroglial phenotype is critical to providing the cellular context for promoting either Wnt7 activity and co-option or VEGF and angiogenesis. OPCs act in cooperation with reactive astrocytes, which are a source of pro-angiogenics such as angiopoietins 1 (Ang-1) and 2 (Ang-2) and VEGF [28].

Oligodendrocyte precursors (OPCs), expressing oligodendrocyte transcription factor 2 (Olig2), can serve as tumor progenitors in glioblastoma and may be actively involved in white matter vascularization [58]. In OPCs, hypoxia induces autocrine activation of the Wnt7 signaling pathway, which results in endothelial proliferation and extensive OPC migration along the vasculature [59,60]. This process, which is physiologic during the embryological development, may recapitulate in adults by the influence of the TME. The use of transgenic lines that differentiate among glial populations showed divergences between Olig2+ OPCs and Olig2− cells conducts. Olig2+ OPC cells utilize vascular networks as a pathway to move over time, and this OPC–vascular relationship is mediated by Wnt7 signaling, essential for brain angiogenesis. Conversely, Olig2− cells (expressing glial fibrillary acidic protein, GFAP+) tend to associate along the vascular walls and are required for normal vessel development. In fact, the ablation of GFAP+ cells causes irregular vessel development and a reduction in vascular endothelial growth factor (VEGF) expression [61]. Recently, it has been described that OPC-like (OPCL) cells in glioma express Wnt7 and invade the brain via single-cell vessel co-option [62]. In a practical sense, the gene targeting of Wnt7a/7b or the pharmacological inhibition of Wnt in experimental models, prevented vessel contact of OPCL with glioma cells. In contrast, Olig2− cells show a collective perivascular migration and enhanced VEGF signaling that increased and distorted the tumor vasculature and caused BBB leakage, leading to microglial activation and macrophage infiltration.

Although the former correlation has not been conducted to date, it might be sensible to assume that angiogenesis and vascular co-option occur in the early stages or in the moderately aggressive glioblastoma types or regions, where the perivascular niche is set around a vessel with microvascular sprouting or vascular cluster conformation. In contrast, highly proliferative tumors or exceptionally necrotic areas rely on vascular mimicry and transdifferentiation, leading to a clustered vascular pattern with garland or glomeruloid conformation.

A decisive repercussion of aberrant neovascularization is the alteration of the BBB. Hyperpermeability leads to interstitial edema and extravasation of plasma proteins and immune components [35]. Besides, the increase in interstitial pressure alters the blood flow and restricts oxygenation and leukocyte recruitment. Ischemia, in turn, leads to HIF-1α secretion and results in new vessel formation [39], thereby constituting a deleterious vicious circle.

## 5. Endothelial–Tumor Cell Crosstalk

To understand the endothelial–tumor interrelation, one must be aware of the perivascular niche components and dynamic transformations. The interaction between tumor cells and the endothelium is bidirectional and somewhat synergistic. Surprisingly, these two cell types seem to be interchangeable in their roles, responding to demands in the microenvironment [63].

GSCs reside in close connection to the vascular endothelium [10] and promote angiogenesis by releasing proangiogenic factors and chemokines (VEGF; platelet derived growth factor, PDGF; stromal derived factor-1, SDF-1 and transforming growth factor-β, TGF-β). These molecules stimulate endothelial spread and remodelation; they also attract pericytes to conform the neurovascular unit [33] and modulate the immune response [41].

Under severe conditions, neovessel formation may rely on GSC differentiation into an endothelial-like phenotype. As seen in vascular mimicry, extreme hypoxia induces the intratumor formation of pseudocapillary structures, based on a matrix structure and GSC-derived endothelial-like cells [64]. The transformation of tumor stem cells into endothelial-like cells seems to be mediated by epigenetic activation of the wtn5a pathway [65]. The endothelial-like cells, in turn, recruit existing endothelial cells to favor perivascular satellitosis, a recurrent niche conformation responsible for the invasive growth of glioma cells away from the primary tumor [65]. Not only is this transdifferentiation phenomenon important to explain tumor recurrence, but it has also been reported as a cause of anti-VEGF therapy failure [63], as will be discussed later.

Vascular co-option is indeed another process requiring intense cell-to-cell crosstalk. The tumor cell spread along the vessels involucrates three processes: first, the dissolution of the extracellular matrix and its endothelial attachments (VE-cadherin, occludin) and second, the tumor adhesion to the endothelium, by the expression of integrins, neural cell adhesion molecule 1 (L1CAM), CXC chemokine receptors (CXCR), and Epithelial Growth Factor Receptor (EGFR) [55,66,67,68,69]. Simultaneously, endothelial cells attract tumor cell adhesion by secreting chemotactic molecules, such as interleukin 8 (IL-8) [70]. Third, the tumor cells acquire a mesenchymal-like phenotype, rendering migratory capacities [71].

Cell-to-cell crosstalk can be mediated by several means of communication. The paracrine action of locally secreted growth factors and chemokines is a well-characterized one. Yet, novel communication cues have been noted, such as extracellular vesicles and tunneling nanotubes. Extracellular vesicles [72] are secreted by glioma cells and contain essential information in the form of proteins, RNA, DNA, or metabolites; they act locally over the endothelium but have also been detected distally, in plasma and cerebrospinal fluid. Tunneling nanotubes [73] are direct intercellular connections based on actin protrusions between non-adjacent cells, allowing for fast signaling and transport over long distances.

All in all, understanding how the tumor cells drive and transform the endothelium to benefit their growth and infiltration is a key cancer hallmark to address with novel therapies and an intricate interplay to emulate in preclinical models.

## 6. Signaling Pathways Involved in Neovascularization

Several signaling pathways have been described in vascular formation and other related processes of glioblastoma development. Most of them are propelled by proangiogenic factors acting over the tyrosine kinase receptors at the endothelial membranes. The transcendence of these pathways in glioblastoma progression promotes these pathways as plausible treatment targets (Table 1).

(i) **Vascular endothelial growth factor (VEGF)** [81] is the paramount signaling molecule in angiogenesis, particularly the VEGF-A isoform. VEGF secretion is induced under hypoxic conditions by HIF-1α. VEGF can act alone or in synergic collaboration with other proangiogenic factors, such as fibroblast growth factor 2 (FGF-2) and platelet-derived growth factor BB (PDGF-BB) [82]. VEGF stimulates endothelial cells to breakdown the extracellular matrix, to proliferate and migrate, and it also increases the vascular permeability.
(ii) **Fibroblast growth factor (FGF)** [83], in the early stages of angiogenesis it mediates proteolysis of the matrix and endothelial migration; in the later stages, it favors capillary conformation and basal membrane synthesis.
(iii) **Platelet-derived growth factor (PDGF)** acts locally, by stimulating endothelial proliferation, and distally, by inducing extramedullary hematopoiesis.
(iv) **Transforming growth factor β (TGF-β)** [31,84] acts over the extracellular matrix, facilitating its dissolution and also its segregation, in the early and late phases of angiogenesis, respectively. TGF-β also promotes GSC differentiation to pericytes to support neovessel conformation.
(v) **Hepatocyte growth factor/scatter factor (HGF/SF)** [85] directly promotes endothelial proliferation, migration, and organization into capillary tubes. Indirectly, HGF/SF promotes angiogenesis by upregulating VEGF and suppressing thrombospondin 1 (TSP-1).

All five growth factors depicted above are overexpressed in glioblastoma, and their levels of expression have been correlated with worse clinical prognosis.

(vi) **Angiopoietin 1 (Ang-1)** [86] induces vessel formation and stabilization by interacting with the endothelial cell kinase 2 (Tie-2) receptor at the tunica interna.
(vii) **Angiopoietin 2 (Ang-2)** [51,87] also acts on the Tie-2 receptor, but with opposing effects. Ang-2 favors VEGF-mediated endothelial proliferation and migration. Paradoxically, in the absence of VEGF, Ang-2 is an antagonist of Ang-1 and thus promotes tumor vessel regression and leakiness.
  Initially, during the tumor development, co-opted vessels express both Ang-2 and Ang-1, which favors pericyte recruitment and vessel integrity. However, as the tumor progresses, the upregulation of Ang-2 leads to vessel destabilization and disruption. In the presence of Ang-2, VEGF promotes endothelial proliferation and migration, resulting in angiogenesis. The final stages of angiogenesis involve capillary morphogenesis, mediated largely by integrins [88], and the endothelial cells secrete PDGF, which recruits pericytes to the new vessel.
(viii) **Angiopoietin 4 (Ang-4)** [89] is a strong proangiogenic that exerts its function by upregulating the transcription of VEGF.
(ix) **Matrix metalloproteinases (MMPs)** [90] degrade the extracellular matrix but also facilitate the proliferation and migration of pericytes for vessel stabilization.
(x) **Stromal-derived factor-1α (SDF-1α)** [91] is a hypoxia-induced factor that recruits bone marrow-derived CXCR4+ endothelial and pericyte progenitors to form new vessels. Both the SDF-1a/CXCR4 and the ANG-2/TIE-2 pathways seem relevant in promoting vasculogenesis.
(xi) **Sphingosine-1-phosphate (S1P)** [92] is a pleiotropic angiogenic factor, regulating both the early and the late stages of angiogenesis. It drives endothelial proliferation, migration and tubular vessel conformation. It is secreted by endothelial cells and is increased by the presence of glioma cells.
(xii) **Caldesmon 1 (CALD1)** [93] modulates actin filaments of the cytoskeleton, controlling cell morphology and movement. CALD1 expression is augmented in both neoplastic and vascular cells, and it confers worse clinical outcomes.
(xiii) **Annexin A2 (AnxA2)** [94] is a ubiquitous membrane-binding protein whose function varies according to its location. During inflammation, AnxA2 limits vascular permeability by maintaining cadherin endothelial junctions; it modulates the inflammasome, and it enables angiogenesis and tissue healing. However, sustained inflammation might lead to AnxA2 upregulation and excessive pathological angiogenesis [95].
(xiv) **Chemokines and pro-inflammatory factors** are also overexpressed in the tumor microenvironment and have been shown to play an essential role in the neovascularization processes. Importantly, these molecules act as interconnectors between the vascular and immune tumor responses [96]. Among chemokines, IL-8 is a paradigmatic example, acting over two cell-surface G-protein-coupled receptors (CXCR1 and CXCR2). Endothelial cells secrete IL-8 and other chemotactic factors to activate GBM invasion and vascular mimicry [52,97]. Chemokines have also been implicated in the tumor resistance to anti-angiogenic drugs. For instance, Vatalanib, a small molecule protein kinase inhibitor, collaterally increases the expression of various chemokines and their receptors at the invasive front of GBM; the subsequent increase in myeloid cells and activated endothelial cells could be responsible for the resistance to the anti-angiogenic effect of Vatalanib [98].

## 7. Perivascular Satellitosis

Perivascular satellitosis is the result of the vessel co-option process. From a histological standpoint, glioblastoma cells infiltrate the surrounding brain in four different ways, namely by individual cell migration, collective cell invasion, perineuronal satellitosis, and perivascular satellitosis [99]. Perivascular migration, by means of co-option, is responsible for microscopic tumor extension beyond the surgical resection margins, contributing to the high local recurrence rates. In addition to Ang-2, CXCR4/SDF-1α and IL-8, there are other specific signaling molecules which are probably responsible for cell migration along the vessels [52]. In concrete, bradykinin and EGFRvIII, a mutational isoform of EGFR, are involved in tumor cell migration [69]. Cdc42 promotes the formation of actin cytoplasmic extensions (flectopodia) to modulate pericytes adhesion and facilitate tumor migration [100].

Interestingly, the glial phenotype of the tumor might determine its tendency to individual-cell or collective-cell invasion along the vessels. In view of the fact that the Olig2-Wnt7a signaling axis induces individual-cell co-option [101], it has been suggested that oligodendrocyte-like glioblastomas (Olig2+) spread in a subtle, single-cell form and preserve the BBB. Meanwhile, astrocyte-like glioblastomas (Olig2-) extend in a collective-cell way, disrupting the BBB and causing marked inflammation and edema.

## 8. Sustained Vascularization and Immunomodulation

Sustained vascularization and immunomodulation are two interconnected hallmarks of cancer ascribed to glioblastoma [96]. The reciprocal communication between the vascular and the immune systems has many connotations. To begin with, the resident microglia and tumor-associated macrophages (TAMs) promote vascularization by the overexpression of multiple pro-angiogenics; of special relevance is the CXCL2 pathway [102]. Immunosuppressive M2-TAMs predominate in tumor hypoxic regions and have proangiogenic activities. The link between hypoxia and the immunosuppressive microenvironment is pivotal in promoting tumor angiogenesis.

On the other side, VEGF holds immunomodulation properties. VEGF is capable of inducing chemotaxis and a phenotype switch in microglia, from an M1 pro-inflammatory to a M2 anti-inflammatory stage. [103]. Furthermore, VEGF can attract regulatory T cells and myeloid-derived suppressor cells [104]. Ang-2 increases neutrophil infiltration and promotes its adhesion to the tumor endothelium [105]. On the other hand, TGF-β increments regulatory T cells that inhibit the cytotoxicity and induce T cell apoptosis [106].

This complex interplay is still under investigation, but it holds promise for the combination of multifactorial treatments.

## 9. Preclinical Models

The traditional two-dimensional cell cultures have been surpassed by three-dimensional models, owing to their power to simulate tissue distribution, cell-to-cell interplay, and BBB features. For decades, the introduction of microvascularization into these preclinical designs has been a hurdle. Yet, the inability to replicate such an essential characteristic of glioblastoma has impacted the accuracy of the models for drug assessment and our understanding of microenviroment interactions and the regulation of angiogenic pathways. To overcome this limitation, several tactics have been proposed, which will be discussed in the following lines.

### 9.1. BBB Spheroid Models

Spheroids are composed of cell aggregates obtained by cell culture in a low-adherence medium or by means of the hanging drop technique. BBB spheroids can be constructed by co-culturing astrocytes in the center of the sphere, surrounded by a layer of pericytes and an outer coverage of endothelial cells [107]. The capillary network remains superficial and scarce; so, as the spheroid increases in size, a necrotic core evolves inside [108]. The permeability of the BBB in spheroids seems comparable to in vivo animal studies but does not accurately reproduce human transmembrane transport regulations [109].

In order to directly study the interactions between endothelial and tumor cells, McCoy et al. [70] designed a layered model in which glioblastoma cells were seeded on the top of a collagen-based matrix, and the endothelial cells lay on the bottom. The authors were indeed able to demonstrate the synergistic crosstalk between these two cell types. 

### 9.2. Vascularized Organoids

Organoids are another three-dimensional structure that better recapitulates the heterogeneity of a tissue [110]. They are obtained by culturing pluripotent stem cells in specific mediums that promote cell differentiation. By sequentially changing the medium, various cell subtypes emerge, self-organize, and assemble into a tissue structure. However, they require a long formation time and, even then, a complete organ maturation is hardly ever achieved [111].

Vascularization can be induced in brain organoids by co-culture with endothelial cells and treatment with VEGF to facilitate vascular maturation and reproduce BBB properties to some extent [112,113].

### 9.3. Perfusable 3D Models (Cancers-on-Chip)

Perfusable models are dynamic models that try to mimic the blood flow, using a pulsatile pump or exploiting gravity or capillary effects [114]. Microchannels are carved in the matrix, usually by microneedle insertion, and then, fluid is allowed to circulate along them. The movement of fluid recreates shear stress over the vessel wall, which is indeed an important regulator of permeability [115]. The main defect of these models is the vessel diameter, which exceeds by far the dimensions of the human microvasculature [97].

### 9.4. Matrix-Based Models

A matrix of natural or synthetic components serves as an extracellular matrix, an integral, yet frequently disregarded, component of the glioblastoma tissue. Varying the matrix composition and biomechanical properties gives this type of model diversity and versatility [116]. For instance, collagen I microfibers reach a stiffness and a Young’s modulus very close to the human brain; adjusting this density helps with cell migration, adhesion, and infiltration, as well as with vasculogenesis processes. Another example is hyaluronic acid scaffolds, a constitutive component of the natural extracellular tissue, which could result in a more physiological milieu for replicating the glioblastoma cell behavior [117].

### 9.5. Bioprinted Models

Tissue printing permits an adequate control of cell distribution and is a highly reproducible technique [97]. In fact, vascularized bioprinted models have been conceived where the co-culture of endothelial cells with patient-derived glioblastoma cells led to a generously vascularized tissue and induced a migratory phenotype of tumor cells [118]. Yi et al. [119] were able to recreate an oxygen gradient similar to the glioblastoma ecosystem and applied it for drug combination therapy testing. Their glioma-on-chips, printed with patient-derived cancer cells, reproduced patient-specific sensitivity against different chemoradiation combinations.

### 9.6. The Chick Embryo Chorioallantoic Membrane (CAM)

The chick embryo CAM assay to study angiogenesis has been used to implant glioblastoma, either onto the CAM surface or injected in the CAM circulation [120]. Moreover, it is also possible to investigate the anti-angiogenic potential of different molecules used as anti-angiogenic drugs in the adjuvant treatment of tumors [121]. All these studies have confirmed the utility and versatility of the CAM assay to study the tumor progression of human glioblastoma.

### 9.7. Major Challenges in Model Development

The inaccuracy in reproducing the whole complexity of the human brain harvesting the glioblastoma microenvironment, along with the low replicability rates, is the major limitation of the current preclinical models. The advancement in the understanding of brain physiology and tumor cell interactions translates into more elaborate and dynamic constructs. In this context, human-induced pluripotent stem cells have become an important source of neural cells for 3D models; nonetheless, cell differentiation methods are time-consuming and expensive, and obtaining stable and reliable neural cell lines remains arduous. In addition, identifying the chief properties of every model is a pending task that could dictate the most appropriate application for each of the modes. Finally, patient-customized designs are the ultimate goal in view of the prospective applications of personalized medicine.

## 10. Anti-Angiogenic Therapeutic Approaches

Excessive angiogenesis and altered vessel function are the two main hallmarks of glioblastoma; therefore, anti-angiogenic therapies were postulated as a well-founded strategy against this tumor. Many anti-angiogenic therapies targeting the VEGF pathway are currently undergoing phase II/III trials for brain tumors. These include monoclonal antibodies against VEGF-A (bevacizumab), tyrosine kinase inhibitors against VEGFR-2 (cediranib, sunitinib, and vandetanib), and soluble decoy receptors developed from VEGFR-1 that selectively inhibit VEGF activity (aflibercept). Unfortunately, to date, anti-angiogenic drugs have shown hardly any benefit on patient outcomes.

The increasing comprehension about the TME and the chronology of the evolution of the perivascular niche, the relation between the tumor cells and the endothelium, and the interconnection between the vascular and the immune systems, all this collective knowledge has made it possible to understand the reasons for the previous unsuccessful treatment attempts and has encouraged the development of novel anti-angiogenic regimens.

### 10.1. Bevacizumab: State of the Art and Reasons for Failure

Bevacizumab was the first anti-VEGF drug approved by the American Food and drug administration (FDA) and the only one recommended to date in glioblastoma treatment [122,123]. The encouraging results of various phase II trials (radiological response rates of 21–61% for recurrent GBM and 6-month free survival rates of 30–51%) [124], led to the introduction of bevacizumab (alone or in combination with irinotecan) for recurrent GBM. A remarkable effect of bevacizumab is the reduction in peritumoral edema and the subsequent dismissal of steroids, which significantly increases the quality of life [125]; this is attributable to vascular normalization and not to the antitumor effect per se.

Still, bevacizumab provides only transient benefit and, sooner rather than later, the tumor recurs, and it does so in a highly infiltrative form, resistant to other therapies [6,7,8]. Several explanations have been proposed, based on TME modifications and escape tactics. In line with the radiologic appearance of the recurrences, two distinct escape mechanisms have been proposed [126]: the proangiogenic mode, in which glioblastoma potentiates alternative proangiogenic pathways independent of VEGF, and the proinvasive mode, in which the tumor sacrifices neovascularization in favor of distal invasion.

The proangiogenic response might rely on activation of other kinase-mediated pathways, such as PDGF-C [127] or SDF-1α [90], to bypass VEGF and induce angiogenesis or vasculogenesis. Moreover, vascular mimicry [128] and tumor stem cell transdifferentiation [63,88], both constitutively VEGF-independent processes, have also been pointed to as potential escaping mechanisms. Both the mesenchymal transition and the vessel co-option events are probably mediated through the kinase mesenchymal–epithelial transition factor receptor (MET) in a hypoxia-dependent manner [129]. In parallel, glioma-associated macrophages are stimulated by glioma cells to produce tumor necrosis factor-α (TNFα), which activates the endothelial cell expression of the proangiogenic molecules (VCAM-1, ICAM-1, CXCL5, and CXCL10). It has been proven that high TNFα levels correlate with worse response to bevacizumab [130].

By means of proinvasive evasion [131,132], tumor cells leave the hostile nonvascular microenvironment towards healthy tissue, using the normal vasculature as a railway. Vascular co-option, a VEGF-independent process, experiences a relative increase after anti-VEGF treatment [114]. The HGF/c-MET axis is postulated as the key mediator of this escape response [133]; this is a tyrosine-kinase-initiated pathway, active under hypoxic and non-hypoxic conditions, leading to endothelial activation, tumor cell proliferation, invasion, and resilience. The induction of MMPs, which degrade the extracellular matrix and facilitate invasion, has also been implicated in anti-VEGF drug resistance [134]. Adding some complexity, metabolic reprogramming is also an adaptative response, a Warburg effect-mediated mechanism that increases tumor cell survival and proliferation in low-glucose mediums [78].

### 10.2. Novel Anti-Angiogenic Therapies

Over the last decade, several innovative anti-angiogenic strategies have been designed, either to try to overcome bevacizumab resistance or as a completely independent plan.

**Targeting kinase signaling pathways**. As previously mentioned, most proangiogenic factors act over the tyrosine-kinase receptors of endothelial cells, and these receptors or the intracellular transductors can become objectives of treatment. For instance, blockage of the PI3K/AKT cascade, implicated in tumorigenesis, is being tested as a single agent (perifosine, phase II trial) or in combination with bevacizumab (NVP-BKM12, phase II trial), with preliminary positive results [129]. Cabozantinib, a dual inhibitor of MET and VEGFR-2, has been tested in patients who are refractory to bevacizumab, with a modest survival increase in a phase II trial.

**Targeting alternative proangiogenic factors**. In addition to VEFG, other proangiogenic molecules are potent tumor promoters, as is the case with Ang-2. Trebananib, a fusion protein that sequesters Ang1/Ang2, seemed a potential candidate for treatment. However, it failed to provide benefit and even conditioned deleterious responses [135], something that emphasizes that not all targets are appropriate in the myriad processes of angiogenesis. 

**Combination of anti-angiogenics with hypoxia activated prodrugs**. Conceptually, hypoxia-activated prodrugs are quiescent chemotherapeutics that become active after metabolization in hypoxic regions. A complex theoretical strategy uses redox-responsive nanocarriers for delivering a combination of an anti-angiongenic and a hypoxia-activated prodrug [136]. In this combination, the first components are meant to enhance the delivery of the latter, which, once transformed, is the actual anti-tumor molecule.

**Oncolytic viruses**. Although still in preclinical phase stages, the use of oncolytic viruses is a clever anti-angiogenic postulation. The virus selectively infects the glioblastoma cells and either induces cell death at the completion of the replication cycle or serves as a vector to deliver therapeutic transgenes. For instance, an oncolytic virus armed with IL-15 provided anti-angiogenic capacity by reducing VEGF in glioblastoma cell lines [112].

**Metformin**. Although modern drug engineering is the mainstream, the potential anti-angiogenic effect of commonly used substances remains a field to exploit. Metformin, an ordinary antidiabetic, might hide a not-so-conventional anti-tumor effect, even that of hampering angiogenesis [137]. Although it seems a safe adjunct to combination therapies, it has yet to demonstrate clinical efficacy.

### 10.3. Vascular Normalization and Immune Checkpoint Inhibitors

In glioblastoma, the inherent difficulty of drugs to traverse the BBB might be aggravated by the previously specified vascular irregularities. The inefficient drug delivery, even when the tumors are over-vascularized, has been regarded as a consequence of unregulated angiogenesis. From this has emanated the idea of vessel normalization, i.e., using anti-angiogenic agents to trim, instead of destroy, the tumor vasculature so as to improve the delivery of drugs [138]. Nonetheless, the normalized state lasts for a limited time, after which neovascularization reappears. The so-called normalization window remarks the importance of timing and dosing in combination therapies [138].

A less hypoxic and functional vasculature is also a requisite for leukocyte migration. Vascular normalization transforms the immunosuppressive microenvironment into an immune-stimulatory one, increasing T-cell infiltration [139]. The idea of combining vascular normalization and immune checkpoint inhibitors assumes that alleviating the harsh microenvironment (immunosuppressive, abnormal vascularization, and hypoxia) could improve the already welcoming results of immunotherapy.

So far, this combination has been tested in preclinical animal models, with favorable outcomes. For example, using of a mix of peptides that bind to abnormal tumor blood vessels, He et al. [140] achieved angiogenic vessel remodeling and edema alleviation in a murine model. Less leaky and activated vessels reduce hypoxia and enhance lymphocyte infiltration. Moving one step further, Di Tacchio et al. [141] employed a combination of anti-angiogenics (blocking VEGF and Ang-2) plus an immune checkpoint inhibitor (PD-1) in an orthotopic glioblastoma model. This synergistic mix significantly increased survival, suggesting that vascular normalization and alleviation of immunosuppression could be a prerequisite for successful immune checkpoint therapy. On a similar line of thought, Song et al. [142] treated glioblastoma mice with VEGF-C and observed increased lymphangiogenesis in the meninges, with a robust T cell migration from cervical lymphatic ganglia towards the brain tumor. VEGF-C also provided benefits in combination with anti-programmed death-1 therapy (anti-PD-1). The study suggests that inducing T cell priming by VEGF-C enables the efficient effect of checkpoint inhibitors in tumors confined to the central nervous system.

### 10.4. Clinical Trials of Anti-Angiogenic Drugs

Several clinical trials have been carried to test the effectivity of anti-angiogenic therapies as an alternative or as a complement to conventional oncological treatments. Table 1 compiles a selection of finished or ongoing phase II/III clinical trials of anti-angiogenic therapy for glioblastoma. Most of these trials target the VEGF pathway, either with the monoclonal antibody against VEGF-A (bevacizumab, marizomib) [75,124] or with small molecule tyrosine kinase inhibitors against VEGFR-2 (donitinib, sunitinib) [74,79]. Other modern strategies include combination regimens, where bevacizumab is combined with other anti-angiogenics (bevacizumab + trebananib, a sequester Ang1/Ang2) [135], or with a myriad of immune-based approaches, including oncolytic viruses (VB-111 + bevacizumab) [76] and anti-PD1 (camrelizumab + bevacizumab) [80]. Although the outcomes in some currently ongoing trials are pending, to date no definitive benefit has been reported with the use of anti-angiogenics in the clinical setting.

## 11. Future Directions

Prospective research should address the two main unmet goals regarding the vascular microenvironment in GBM. First is the inability of the current bioengineering methods to replicate the delicate tumor neovascularization and vascular exchange properties. The 3D models must be upgraded to account for the BBB behavior in the physiologic and disruptive stages. These models should also include all the components of the neurovascular unit, as well as the associated signaling molecules that drive intercellular communication. The standardization of the model composition and reproducibility of the cellular in vitro differentiation ought not to be disregarded.

Second is the inefficacy of anti-angiogenic therapies or, in the best of the cases, their fleeting clinical effects. The combination of therapies that target neovascularization processes with conventional chemotherapeutics or immunotherapies is an ingenious option. Moreover, the use of anti-angiogenics as a means to normalize tumor vasculature and ease the action of other treatment regimens deserves further exploration in both preclinical and clinical studies. Most of all, a global effort should be made to increase the number of research laboratories allocated to GBM, which are essential in the process towards successful clinical trials.

## 12. Conclusions

Today, glioblastoma still represents a devastating disease with defeated treatment options. The heterogeneous intratumor genetic subpopulations, the infiltrative nature, the uneven vascularization patterns, and the dynamic capacity to adapt and resist internal or endogenous aggressions have been blamed for the treatment inefficacy. Most of these properties derive from the unique interaction between the tumor and the brain microenvironment. Studying these interactions has brought fresh and promising curing strategies, such as the synergic combination of anti-angiogenics and immunotherapy. In the near future, we will see innovative neuroimaging able to assess the molecular profile and the perivascular niche status; we expect that customized in vitro models will become available for testing patient-specific regimens, and we anticipate that precision medicine will effectively address and control glioblastoma in all its complexity.

## Figures and Tables

**Table 1:** Selected phase II and III, finished or ongoing, clinical trials of anti-angiogenic therapy in glioblastoma, alone or in combination with other treatments.

Clinical Trial Reference	Intervention	Mechanism of Action	Patient Population	Design	Primary Endpoint	PFS	Conclusion
Sharma et al. 2019 [74]	Dovitinib	Tyrosine-kinase receptor inhibitor	Recurrent or Progressive GBM	Phase II, non-randomized, parallel	PFS 6 months	PFS-6 12% in anti-angiogenic naïve and 0% in anti-angiogenic exposed	Dovitinib was not efficacious in recurrent GBM
Bota et al. 2021 [75]	Marizomib	Panproteanoma inhibitor + monoclonal antibody anti-VEGF	Recurrent or Progressive GBM	Phase II, non-randomized, intra-patient dose escalation	PFS 6 months	PFS-6 29.8% OS 9.1 months	No benefit of the addition of marizomib to bevacizumab
Cloughesy et al. 2020 [76]	VB-111 + bevacizumab	Oncolytic virus + anti-VEGF	Recurrent GBM	Phase III	OS	OS 6.8 months	No benefit and higher rates of adverse events
Brenner et al. 2021 [77]	Evofosfamide + bevacizumab	hypoxia activated pro-drug + anti-VEGF	Recurrent GBM	Phase II single-arm	Safety	PFS-4 31% OS 4.6 months	Deserves further investigation
Lee et al. 2021 [78]	Trebananib + bevacizumab	Sequester Ang1/Ang2 + anti-VEGF	Recurrent GBM	Phase II randomized	PFS 6 months	PFS-6 22.6%	Detrimental (lower PFS than bevacizumab alone)
STELLAR NCT03025893 [79]	Sunitinib	Tyrosine-kinase receptor inhibitor	Recurrent GBM	Phase II/III randomized, against lomustine	PFS	Ongoing	-
NCT04952571 [80]	Camrelizumab + Bevacizumab	Anti-PD1 + anti-VEGF monoclonal antibodies	Recurrent GBM	Phase II non-randomized, parallel	PFS	Ongoing	-

GBM, glioblastoma; OS, overall survival; PFS, period of free survival.

## Data Availability

Data sharing not applicable.
